# Primary penile Kaposi’s sarcoma in HIV-seronegative patient: a case report and literature review

**DOI:** 10.1590/S1677-5538.IBJU.2020.05.03

**Published:** 2020-07-31

**Authors:** Gianmartin Cito, Roberto Di Costanzo, Simone Morselli, Andrea Cocci, Raffaella Santi, Gabriella Nesi, Alessandro Natali, Andrea Minervini, Marco Carini, Fabrizio Travaglini

**Affiliations:** 1 University of Florence Careggi Hospital Department of Urology Florence Italy Department of Urology, Careggi Hospital, University of Florence, Florence, Italy; 2 University of Florence Department of Health Sciences Florence Italy Pathology Section, Department of Health Sciences, University of Florence, Florence, Italy

## INTRODUCTION

### Background

Kaposi’s Sarcoma (KS) is a reticuloendothelial system tumor, that may involve the skin, mucosa and viscera (1). It can be considered a malignant vasoformative neoplasia with endothelial proliferation and spindle cell formation on histologic examination. In recent years, there have been several changes in our understanding of KS, including its evolving epidemiology, pathogenesis, new clinical presentations and associations, descriptions of new histologic variants, and the emergence of novel biomarkers with promising targeted therapeutic agents (2). Despite these advances, KS remains the most prevalent malignancy among patients with acquired immune deficiency syndrome (AIDS), being related with drugs or transplant-associated immunosuppression. To our knowledge, this disease has a tight link to Human Herpesvirus 8 (HHV-8) infection, also known as KSHV (Kaposi Sarcoma-associated Herpes Virus). KS can occur in five different epidemiologic-clinical settings: AIDS-related (also known as epidemic), iatrogenic (iatrogenic immunodeficiency, such as that seen in organ transplant recipients), endemic (commonly in sub-Saharan Africa in individuals seronegative for human immunodeficiency virus, HIV), Classic (also known as sporadic KS) and MSM (man who have sex with man) without HIV infection, who are young or middle aged, not immunocompromised (3, 4). The epidemiology suggests that this cancer had an origin independent of HIV, as well as a directed search of DNA led to the discovery of KSHV involvement in the pathogenesis of KS (5). Actually, it is known that a combination of KSHV infection and impaired host immunity might be responsible for KS. However, although AIDSrelated KS and iatrogenic KS are associated with welldefined immunodeficiency, the impaired immune function in classic KS (related to ‘immunosenescence’, as an ageing immune system) and endemic KS (related to chronic infection and malnutrition) is not exactly characterized. In addition, KSHV can cause: I) two lymphoproliferative disorders, represented by the primary effusion lymphoma (PEL) (6) and the multicentric Castleman disease (MCD) (7), II) an inflammatory syndrome called KSHV inflammatory cytokine syndrome.

Whit regard to the clinical presentation, each recognized variant has different manifestations and different visceral involvement. It has been estimated that KS confined to the penis is uncommon and is more often observed in patients with AIDS (8), representing the first manifestation of KS in approximately 2 to 3% of HIV-positive patients. Otherwise, up to 20% of these patients may develop genital lesions in the course of the systemic disease (9, 10). Even more rare primary KS of the penis may be in case of HIV seronegative patients.

The aim of this study is to describe an uncommon clinical presentation of genital KS in HIV-seronegative man and to perform a narrative literature review of the cases described to date.

### Epidemiology

KS, first described by Moritz Kaposi in 1,872, is a rare neoplasm that origins from the endovascular cells in a multifocal way-This enigmatic infrequent malignant disease has since received much resonance after the AIDS epidemic in the early 1980s, with an incidence of classic KS ranged from 0.01 per 100.000 personyears for the UK and 0.2 per 100.000 personyears for the USA. However, currently, the incidence of KS is reported to be 200-fold higher in recipients of solidorgan transplants, known as iatrogenic KS form, rather than in the general population (11). The incidence of KSHV in south Africa is very high, reaching >90% in some population, while in Europe prevalence is 20-30%, in Asia and USA is <10% (3). In the Early 1980s with the onset of AIDS emergency, one of the first sign was the rise of KSHV infections. Indeed, a rise of KS incidence of 20.000 time in general population and 300 times in AIDS patients was estimated compared to other immunosuppressed patients (12) with a higher rate for MSM (13). Moreover, with the introduction of combination antiretroviral therapy (cART) the incidence of AIDS related to KSHV decreased considerably (14).

### Physiopathology

KSHV is a large double-stranded DNA herpesvirus with a protein covering by an icosahedral capsid, surrounded by tegument and enclosed in a lipid envelope derived in part from the cell membrane. Different glycoproteins in the viral envelope interact with celltypespecific cellular entry receptors such as integrins (including α3β1, αVβ5 and αVβ3), the cystine-glutamate transporter xCT, heparan sulfate and the tyrosine protein kinase receptor EPHA2). KSHV can infect several different cell types, including endothelial cells, B cells, epithelial cells, dendritic cells, monocytes and fibroblasts. Once inside the cell and after uncoating the virus genome enter in the nucleus where enter in lantecy phaseas episome and undergoes sporadic bouts of lytic reactivation (15). Virus can induce latency in human B cells and endothelial cells, as others Herpes virus. During the latent state expresses the latency locus, which includes ORF71 (who encoding viral inhibitory protein vFLIP), ORF72 (encoding vCyclin), ORF73 (encoding latency-associated nuclear protein (LANA)), ORFK12 (encoding the kaposins, which are signalling proteins) and several microRNAs (miRNAs) (16, 17). The latent genes expressed can promote tumorigenesis supporting the survival of the infected cell. Indeed, vFLIP protein activates I kBκ kinase 1 (IKK1) to stimulate the nuclear factor kBκ (NFκB) pathway to increase cell survival, viral miRNAs inhibit apoptosis. Finally, miRNAs also promote endothelial cell reprogramming, and induces the migration and invasion of endothelial cells and vFLIP promotes vascular proliferation. The reactivation from the latency is determined by different stimuli that are not well defined. During this phase, the virus induces, at first the expression of Immediate early (IE) genes than, Delayed early (DE) genes. Similar to the protein products of latency genes, the protein products of lytic genes can contribute to tumorigenesis. The products of those genes (such as vIL6) can induce proinflammatory and angiogenic factors, including vascular endothelial growth factor (VEGF) and plateletderived growth factor (PDGF) (18, 19). In order to survive and to induce cell survival and cell proliferation, KSHV modulate many host cell signaling pathways, including the phosphoinositide 3kinase (PI3K)-AKT-mTOR pathway, the mitogenactivated protein kinase (MAPK) pathway and the NFκB pathway. KSHV encode also genes with the capacity to inhibit host immune respond. K3 and K5 are lytic genes that encode modulator of immune recognition 1 (MIR1) and MIR2 both of which inhibit major histocompatibility complex (MHC) class I antigen presentation to prevent the immune system. KSHV homologues of interferon regulatory factors (IRFs), viral IRFs (vIRFs), are lytic proteins that inhibit type I interferons. KSHV also encodes three CCchemokine ligands (CCLs, formerly known as vMIPs): vCCL1 (encoded by ORFK6), vCCL2 (encoded by ORFK4) and vCCL3 (encoded by ORFK4.1), which can negatively regulate inflammation. Finally, the KSHV K14 gene encodes for a viral OX2 (vOX2), an immunoglobulin superfamily member with homology to the cellular OX2 membrane glycoprotein (OX2, also known as CD200) that binds to the receptor CD200R and suppressed neutrophil activation, decreased CCL2 (also known as MCP1) and IL8 production and inhibited oxidative burst in neutrophils stimulated to undergo phagocytosis.

### Clinical presentation

The behavior of the disease varies from a singular lesion localized in the skin, to a fleeting extensive respiratory and gastrointestinal visceral involvement. All variants of KS cutaneous lesions usually present as multiple, pigmented, raised or flat, painless that do not blanch. Classic variant (also known as sporadic KS) is typically confined to lower limbs with few lesions. Visceral and mucosal disease is rare and usually occurs in the gastrointestinal tract. Endemic is a typical manifestation of African children often present with multiple lymph nodes with lymphoedema and a very aggressive natural history of the disease, including visceral disease. AIDS-related is characterized by multiple cutaneous lesions on the limbs, trunk and face. Mucosal lesions, such as oral lesion, are common (identified in 20% of patients) and visceral involvement is seen in 15% of patients. Related with Iatrogenic immunodeficiency, such as in organ transplantation. Often presents as cutaneous KS lesions but both mucosal and, rarely, visceral disease can occur. Finally, in MSM patients the clinical manifestations included lesions that can occur at any skin sites, usually few. Visceral and mucosal disease is rare (3). Regarding visceral involvement, organ lesions are uncommon (in one study, only 15% of 469 patients had visceral lesions upon diagnosis with AIDSrelated KS) (20). Gastrointestinal and pulmonary lesions are more present in AIDSrelated KS. Pulmonary lesions present with dyspnea, dry cough and sometimes hemoptysis, with or without fever, are lifethreatening. These lesions typically appear as a diffuse reticule nodular infiltrate and/or pleural effusion on chest radiography. Gastrointestinal lesions are usually asymptomatic, but may bleed or cause obstruction, and their presence is usually confirmed at endoscopy. However, CT scans, bronchoscopy and endoscopy are not warranted in patients unless they present symptoms indicative of visceral lesions (3). When there is clinical suspicion of KS, a biopsy sample is taken to confirm the diagnosis histologically. Pathologic diagnosis can often be made using conventional hematoxylin and eosin (H&E) and it shows some characteristic features such as, vascular proliferation in the dermis, an increased number of vessels without an endothelial cell lining, the presence of extravasated blood, spindle cells express endothelial markers and are considered to be the KS tumor cell (CD34, LYVE1 and VEGF receptor 3) (3). As concerned the therapy in patients with forms of KS when immunosuppression is potentially reversible, the firstline approach is to bolster the immune system. IFNα and alitretinoin (a retinoid panagonist receptor), are approved for AIDSrelated KS, as KSHV directed therapy (3). Otherwise, regarding the management of genital KS, no specific therapy has been described to date.

## CASE DESCRIPTION

**Figures 1 A and B:**
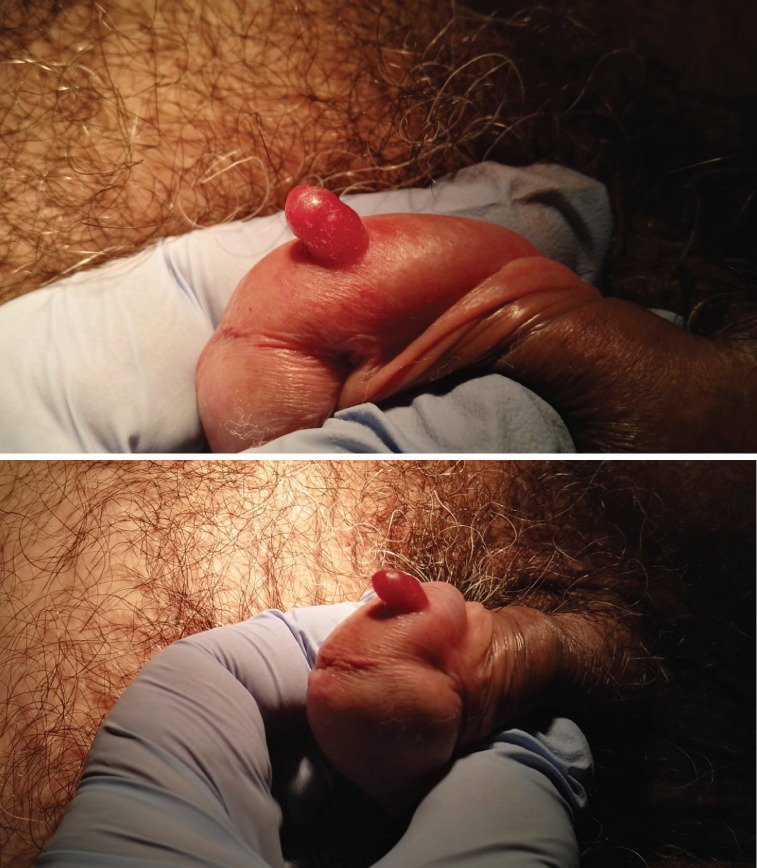
Red painless radish nodule hemangiomalike on the gland near the frenulum.

**Figures 2:**
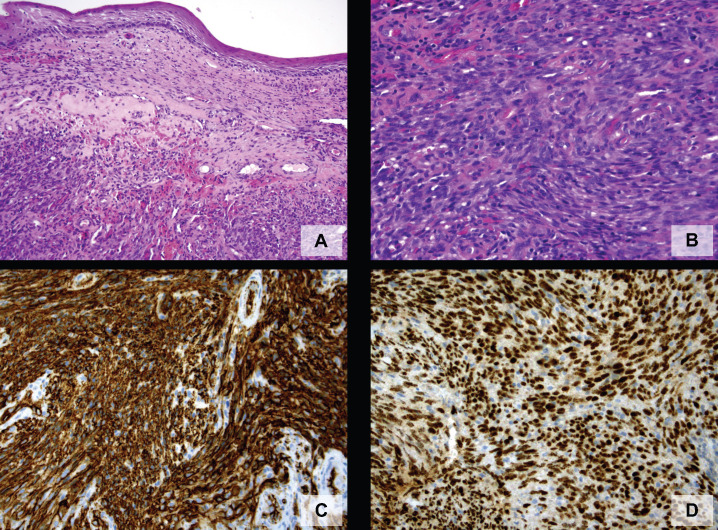
Penile biopsy showed a dermal tumor constituted by intersecting fascicles of spindle cells, arranged around slitlike vascular spaces admixed with numerous extravasated red blood cells and scattered inflammatory cells (A). At higher magnification, spindle cells exhibited mild to moderate atypia (B). Neoplastic cells stained positively for CD34 (C) and HHV-8 (D).

**Figures 3:**
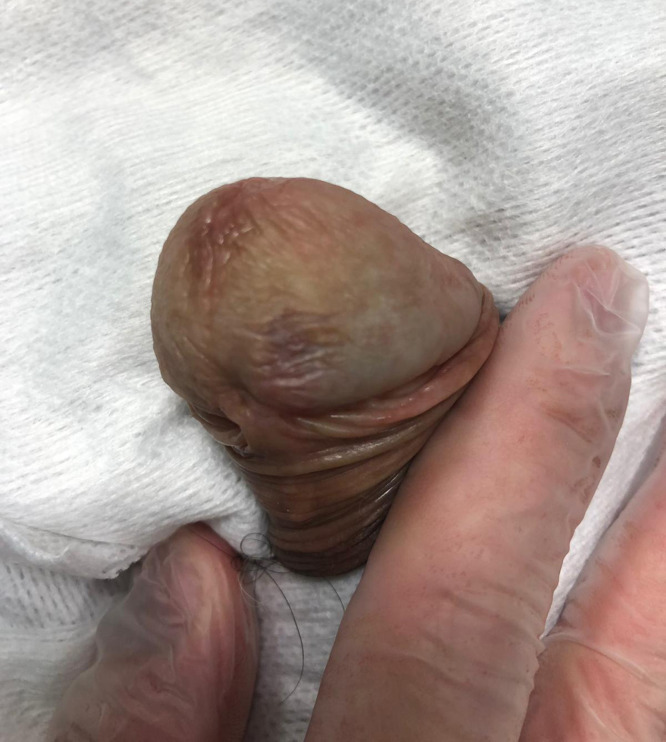
Clinical examination revealed the complete remission of the pathology.

A 71-year-old heterosexual, Caucasian man, referred to our department for the presence of penile neoformation appeared from at least 6 months. At the clinical examination, a 0.6mm x 0.6mm x 0.3mm red painless radish nodule hemangioma-like was found on the gland near the frenulum (Figures 1A and 1B). He did not complain penile bother nor there were palpable inguinal lymph nodes. His past medical history reveled only hypertension and hyperuricemia under treatment. The urine analysis and blood laboratory tests showed normal results. The urine culture was negative for Neisseria Gonorrhoeae, Trichomonas Vaginalis, Ureaplasma Urealitycum, Mycoplasma hominis, Mycoplasma Genitalium, Clamydia Trachomatis. The enzyme-linked immunoassorbant assay (ELISA) sierology was negative for Troponema pallidum and HIV 1-2 infections. A complete surgical excisional biopsy of the lesion was performed, with margin control (Figure-2). The histopathological examination showed a dermal tumor constituted by intersecting fascicles of spindle cells, arranged around slit-like vascular spaces admixed with numerous extravasated red blood cells and scattered inflammatory cells. The immunohistochemical staining evidences for HHV-8 both in the stromal cells and in the endothelial ones. In addition, the spindle cells were positively stained for CD31, CD34, and negatively for AE1, AE3, CITO-B, P63, ACTINA A4. These clinical and histopathological findings were compatible with a typical KS variant. Therefore, computed tomography (CT) of the abdomen and chest was scheduled, not showing any visceral involvement. The 3-months follow-up visit demonstrated the complete remission of the pathology without recurrences (Figure-3).

## DISCUSSION

### Literature review

An English-language literature research was conducted, focusing on the cases of penile KS in HIV positive and negative patients (Table-1). Two different authors (GC. and R. DC.) independently searched Medline, Scopus and PubMed databases using a single query in order to identify all the previous reports describing the diagnosis, clinical presentation, histological findings, therapy and recurrence rate of penile KS. The following terms were included: ((penile) OR penis) AND Kaposi’s sarcoma) AND HIV. Finally, considering the period from 1985 to date, a total of 33 KS cases associated with KSHV, with penis as the only manifestation site of the disease, were found in literature. KS usually affects patients between the fifth and eighth decade of life living on the Mediterranean coastal areas where the HHV-8 infection is widespread. In the KS cases found in literature, patient’s average age was 55.7 years (range 2678 years). As showed by epidemiological evidences that highlight the strong link between the disease pathogenesis and HHV-8 infection, most of the patients with penile KS resulted positive for serology HHV-8 research. Equally, the histopathological examination found typical features of KS (Table-2). Since it was described a high HHV-8 sero-prevalence in individuals with high risk sexual activity, including homosexual, a focus on sexual behaviors are mandatory. However, according to our case, only few patients referred to have risk sexual intercourse (21–23). As concerned to the immunological status, three patients with isolated penile KS reported an immunosuppression HIV related (23, 24). The lesions described are definitely variable for manifestation, (nodule (23, 25–39), papular (22, 34, 40–44), ulcerated (27, 39, 45), granulomatous (21), verrucous (46) dimension 0-5mm (24, 31–33, 35, 40, 42, 43, 47), >6mm (21, 26, 28, 30, 34, 36, 37, 40–42,46), site (gland (21–23, 25, 26, 28–45, 48), coronal sulcus (22, 26, 29, 37, 40, 44, 45), prepuce (24, 47, 49), penilshaft (27, 46, 48), scrotum (23), frenulum (30)), color (reddish (23, 25–27, 29, 32, 34, 36, 40, 44, 45, 49), purplish (23, 29, 30, 33, 39, 41, 42, 43,), bluish (30, 42), skin colored (24), dark brown (48), number (single (26–28, 30–33, 35, 36, 38, 39, 41–43, 45, 47, 49) to multiple (21–25, 29, 34, 37, 40, 44, 46, 48,) and symptomatology (asymptomatic, painfull (45)). The most frequently involved site is the glans, sometimes in associations with swelling and lymphatic edema due to massive involvement and the most common manifestation is a nodular reddish or purplish lesion, single or multiple, sometimes ulcerated too. Lesions may also involve the foreskin, the coronal sulcus, or the meatus. In this last case urinary obstructive symptoms may occur. The involvement of the shaft is rare, usually being related to lesions located on the glans or coronal sulcus. Notably, the lesion observed in our patient was a single red radish pedunculated hemangioma-like lesion of the gland next to the frenulum. To our knowledge, this atypical clinical presentation is similar to others described in literature. Indeed, other comparable lesions described in literature varied from a red purple nodule, or radish 5mm papule in diameter of gland to a 1mm nodule next of the meatus. Nevertheless, it remains a rather infrequent manifestation because of its appearance, that could simulate a benign pedunculated lesion of vessels. According to other cases in literature, our case refers to an immunocompetent HIV-seronegative patient. Therefore, similar cases although rare, could not be infrequent. However, the management of patients with KS should include: exams to exclude ongoing infectious diseases, assessment of patient’s immunological status, histological analysis following surgical biopsy and visceral involvement evaluation through CT or ultrasounds, despite it is not necessary in asymptomatic patients, according to others studies (3). In our case, we managed it with complete surgical excision of the lesion, as described by other authors (24, 26, 28, 30, 31, 35, 36, 38–40, 43, 44, 47, 49), with a disease recurrence in five cases (24, 30, 31, 36, 39) from a period of about 1-2 years. Other approaches described in literature could include radiotherapy (25, 29, 37, 42), subtotal circumcision associated with biopsy (45), cryotherapy associated with 5% Imiquimod cream (21), excisional biopsy associated with IFNα (32), biopsy with chemotherapy (39), CO2 (48), biopsy with radiotherapy (46). Furthermore, in five cases no therapy was performed (22, 23, 33, 34), two of them for the spontaneous regression of the disease (33, 34). The clinical course of primary penile KS is variable and no standardized follow-up exists to date. In general, local recurrences are rare if the primary tumor is completely removed. In one of them (33) the authors refer recurrence of a new penis lesion after seven months, then after one year two lesions on both legs and one on conjunctiva. Other cases of recurrences occurred for therapy with biopsy with IFNα (32) and with CO2 (48). Respect the management of recurrence, it was described only a recurrence after excisional biopsy (24) in which case it was treated with a new biopsy with radiotherapy, with no recurrence. In our patient, at 6-months from surgery there are no signs of disease progression, although it is a too short follow-up period.

**Table 1:** Data of patients with Kaposi’s Sarcoma.

REFERENCE	PATIENT AGE	SEXUAL RISK	IMMUNODEPRESSION	HIV+	HHV	CLINICAL FEATURES
Case of classic Kaposi sarcoma of the penis successfully treated with radiotherapy. Kuriyama, et al. (21)	65	NO	NO	NO	HHV-8	asymptomatic reddish nodules on the glans penis
Kaposi’s sarcoma: An unusual penile lesion in a HIV negative patient. De Rose, et al. (22)	75	NO	NO	NO	HHV-8	painful ulcerated red lesion on the glans that stretched from the urethral meatus to the coronal skin
Topical imiquimod 5% as a treatment for localized genital Kaposi’s sarcoma in an HIV-negative man: a case report. Fairley, et al. (23)	43	YES	NO	NO	HHV-8	two fleshy granulomatous lesions on the glans and corona of the penis, 5-6 mm in diameter
Penile Kaposi’s sarcoma in a HIV negative HHV-8 positive man. Kampantais, et al. (24)	50	NO	NO	NO	HHV-8	0,5 cm in size on the inner layer of the prepuce
Isolated penile Kaposi’s sarcoma in a HIV-positive patient stable on treatment for three years. Lebari, et al. (25)	40	NO	YES	YES	HHV-8	two skin-coloured KS lesions on the prepuce of the penis, 5mm in diameter on the inner layer of the prepuce
Kaposi Sarcoma of the Penis in an HIV-Negative Patient. Cecchi, et al. (26)	52	NO	NO	NO	IgG NEGATIVE PER HHV-8	translucent, domeshaped, reddish nodule on the glans penis near the coronal sulcus. 8 mm in diameter
Primary Kaposi Sarcoma of Penis in HIV Negative Patient. Karami, et al. (27)	47	YES	NO	NO	HHV-8	papular indurate glandular and subcoronal multiple lesions
Kaposi’s Sarcoma of the Penis as an Initial Urological Manifestation of AIDS A Report of Two Cases. Angulo, et al. (28)	28	YES	YES	YES	HHV-8	growing red-purple nodule on his glans penis
Kaposi’s Sarcoma of the Penis as an Initial Urological Manifestation of AIDS A Report of Two Cases. Angulo, et al. (28)	26	YES	YES	YES	HHV-8	multiple cutaneous lesions in the penis, scrotum, right calf and leg
Penile Kaposi’s sarcomas in a circumcised and HIV-seronegative patient. Gonen, et al. (29)	55	NO	NO	NO	not use HHV-8 test on patient	two reddish papules,5 mm in diameter on coronal sulcus near the frenulum and 2 mm in diameter on the glans
Primary Classic Kaposi’s Sarcoma of the Penis in an HIV-Negative Patient. Kim, et al. (30)	68	NO	NO	NO	HHV-8	ulcerated dark reddish nodule on the penile shaft
Isolated Kaposi Sarcoma in two HIV negative patients. Seleit, et al. (31)	34	NO	NO	NO	HHV-8	The nodule was (1x1 cm) in size, on the glans penis lateral to urethral meatus
Exclusive penile Kaposi’s sarcoma: report of an HIV-negative man successfully treated with radiotherapy. Zargari, (32)	71	NO	NO	NO	HHV-8	oedematous penis with purplish macular lesions over the glans penis and a few reddish small nodules on the coronal sulcus
Kaposi sarcoma of the penis in an HIV-negative patient Sarcoma de Kaposi de pênis em paciente HIV negativo. Guevara, et al. (33)	48	NO	NO	NO	HHV-8	The lesion was a purple color papule over the glans near the urethral meatus, measuring approximately 1cm
Kaposi sarcoma limited to glans penis. Conger K, et al. (34)	67	N/A	N/A	N/A	N/A	Single purplish slightly raised nodule (0 10 mm) on the glans near the frenulum
Kaposi sarcoma limited to glans penis. Conger, et al. (34)	55	N/A	N/A	N/A	N/A	Single painless bluish wartlike lesion on the frenulum
Kaposi’s sarcoma of penis. Maiche, et al. (35)	70	N/A	N/A	N/A	N/A	Single nodule (0 5 mm) on the glans; local swelling
Disseminated Kaposi’s sarcoma that is not associated with acquired immunodeficiency syndrome in a bisexual man. Marquart, et al. (36)	44	N/A	N/A	NO	N/A	Single red-brown nodule (ø 5 mm) on the glans
Kaposi’s sarcoma of the conjunctiva. Jaimowich, et al. (37)	74	N/A	N/A	N/A	N/A	Single painless, firm, smooth and purple nodule (ø 5 mm) on the glans near the meatus
Spontaneous healing of Kaposi’s angiosarcoma of the penis. Casado, et al. (38)	77	N/A	N/A	N/A	N/A	Six red smooth papulonodules (ø 3-7 mm) on the glans and inner aspect of the foreskin
Kaposi’s sarcoma of the penis. Zambolin, et al. (39)	47	N/A	N/A	NO	N/A	Single brown pedunculate lesion on the inner aspect of the prepuce near the frenulum
Radiation therapy for classic Kaposi’s sarcoma presenting only on the glans penis. Lands, et al. (40)	54	N/A	N/A	NO	N/A	Multiple blue-purple to brown macules and papules (ø 2-6 mm) on the glans
Radiation therapy for classic Kaposi’s sarcoma presenting only on the glans penis. Lands, et al. (40)	50	N/A	N/A	NO	N/A	Maroon linear growth (8 mm) on the glans
Kaposi sarcoma limited to the glans penis. Myslovaty, et al. (41)	70	N/A	N/A	NO	N/A	Single purplish, slightly raised nodule (ø 5 mm) on the glans
Primary classic Kaposi’s sarcoma of glans penis - appearance on magnetic resonance imaging. Guy, et al. (42)	69	N/A	N/A	NO	N/A	Single smooth reddish-violet nodule on the glans (ø 15 mm)
Purplish penile papule as a presenting sign of Kaposi’s sarcoma. Grunwald, et al. (43)	75	N/A	N/A	NO	N/A	Single, non-tender, purplish papule (ø 5 mm) on the glans
Kaposi’s sarcoma limited to the penis treated with cobalt-60 radiotherapy. Ruszczack, et al. (44)	78	N/A	N/A	NO	N/A	Multiple dome-shaped violaceous and crusted nodules (ø 5-10 mm) on the glans, coronal sulcus and foreskin; massive oedema of distal shaft
Primary Kaposi’s sarcoma of the glans penis. Koyuncuoglu, et al. (45)	52	N/A	N/A	NO	N/A	Single painless nodule on the glans
Adult genitourinary sarcomas: a report of seventeen cases and review of the literature. Berkmen, et al. (46)	55	N/A	N/A	N/A	N/A	Single purplish ulcerated nodule on the glans
Adult genitourinary sarcomas: a report of seventeen cases and review of the literature. Berkmen, et al. (46)	60	N/A	N/A	N/A	N/A	Single purplish ulcerated nodule on the glans
A case of classical Kaposi’s sarcoma of the penis showing a good response to high energy pulsed carbon dioxide laser therapy. Chun, et al. (47)	54	N/A	N/A	NO	N/A	Multiple, dark-brownish plaques on the glans and shaft
Penile Kaposi’s sarcoma preceded by chronic penile lymphoedema. Schwartz, et al. (48)	45	N/A	N/A	NO	N/A	Lymphoedema followed by onset of two verrucous lesions on the glans and on the ventral shaft (ø 30 mm) 2.5 years later
Penile Kaposi’s sarcoma in a human immunodeficiency virus-seronegative patient. Kavak, et al. (49)	43	N/A	N/A	NO	N/A	Two reddish and smooth papules (ø 4 mm) on the glans and coronal sulcus

**Table 2:** Treatment, histopathological findings and recurrence of patients with KS.

REFERENCE	HISTOLOGY	TREATMENT	RECURRANCE	RECURRENCE FEATURE	RECURRENCE THERAPY
Case of classic Kaposi sarcoma of the penis successfully treated with radiotherapy. Kuriyama, et al. (21)	slit-like spaces filled with red blood cells and extensive proliferation of spindle-shaped cells	4-MV X-ray radiotherapy, a total of 60 Gy.	NO	N/A	N/A
Kaposi’s sarcoma: An unusual penile lesion in a HIV negative patient. De Rose, et al. (22)	groups of spindle cells, extravascular erythrocytes, and macrophages filled with hemosiderin	subtotal circumcision and a glans biopsy	NO	N/A	N/A
Topical imiquimod 5% as a treatment for localized genital Kaposi’s sarcoma in an HIV-negative man: a case report. Fairley, et al. (23)	spindle-cell proliferation. High cellularity and mitoses. Vascular spaces and capillaries with some red blood cells entrapped between spindle cells	cryotherapy; At week 8 imiquimod 5% cream for a total of six weeks of treatment.	NO	N/A	N/A
Penile Kaposi’s sarcoma in a HIV negative HHV-8 positive man. Kampantais, et al. (24)	classical Kaposi’s sarcoma	excision	NO	N/A	N/A
Isolated penile Kaposi’s sarcoma in a HIV-positive patient stable on treatment for three years. Lebari, et al. (25)	penile prepuce KS.	excision of the lesion	YES	new skin-coloured lesion at the frenulum of the glans penis, 6X6X3 mm	cryotherapy and 5% imiquimod + surgical excision biopsy
Kaposi Sarcoma of the Penis in an HIV-Negative Patient. Cecchi, et al. (26)	spindle-shaped cells intermingled with vascular slits with intra- and extravascular red blood cells	excision of the lesion	NO	N/A	N/A
Primary Kaposi Sarcoma of Penis in HIV Negative Patient. Karami, et al. (27)	N/A	NO	NO	N/A	N/A
Kaposi’s Sarcoma of the Penis as an Initial Urological Manifestation of AIDS A Report of Two Cases. Angulo, et al. (28)	KS	NO	NO	N/A	N/A
Kaposi’s Sarcoma of the Penis as an Initial Urological Manifestation of AIDS A Report of Two Cases. Angulo, et al. (28)	KS	NO	NO	N/A	N/A
Penile Kaposi’s sarcomas in a circumcised and HIV-seronegative patient. Gonen, et al. (29)	vascular lesions with spindle cell proliferation and increased mitotic activity. Vascular clefts with blood elements. Atypical spindle cells are organized as interlacing bundles with extravascular erythrocytes scattered around	excision of the lesion	NO	N/A	N/A
Primary Classic Kaposi’s Sarcoma of the Penis in an HIV-Negative Patient. Kim, et al. (30)	spindle cells scattered between collagen bundles and small vascular proliferation (CD31-cd34 +)	circumcision	NO	N/A	N/A
Isolated Kaposi Sarcoma in two HIV negative patients. Seleit, et al. (31)	Confirmatory immunohisto. chemical staining for CD 34 antibody was done and reve. aled positive staining for endothelial cells and malignant spindle shaped cells	excision	NO	N/A	N/A
Exclusive penile Kaposi’s sarcoma: report of an HIV-negative man successfully treated with radiotherapy. Zargari, (32)	proliferation of spindle cells forming slit-like structures in the dermis, compatible with typical Kaposi’s sarcoma	radiotherapy with 3000 rad fractionated in 10 consecutive days.	NO	N/A	N/A
Kaposi sarcoma of the penis in an HIV-negative patient Sarcoma de Kaposi de pênis em paciente HIV negativo. Guevara, et al. (33)	proliferation and fascicles of spindle cells associated with angiogenesis		N/A	N/A	N/A
Kaposi sarcoma limited to glans penis. Conger, et al. (34)	N/A	Local excision	YES	Onset of a new lesion on the toe after 1 year	N/A
Kaposi sarcoma limited to glans penis. Conger, et al. (34)	N/A	Local excision	NO	No recurrence after 5 years	N/A
Kaposi’s sarcoma of penis. Maiche, et al. (35)	N/A	Local excision	YES	Local recurrence after 1.5 years; no further recurrences after 3 years	N/A
Disseminated Kaposi’s sarcoma that is not associated with acquired immunodeficiency syndrome in a bisexual man. Marquart, et al. (36)	N/A	Local excision + IFN-	YES	Onset of three new lesions on the toe, the thigh and the knee after 2 years	N/A
Kaposi’s sarcoma of the conjunctiva. Jaimowich, et al. (37)	N/A	Not performed	YES	Spontaneous regression of the primary lesion and onset of a new lesion on the back after 7 months; new lesions on both legs and in the conjunctiva after 1 year	N/A
Spontaneous healing of Kaposi’s angiosarcoma of the penis. Casado, et al. (38)	N/A	Not performed	YES	Spontaneous regression of the primary lesions after 1 year; no recurrences after 1.5 years	N/A
Kaposi’s sarcoma of the penis. Zambolin, et al. (39)	N/A	Circumcision	NO	No recurrences after 10 months	N/A
Radiation therapy for classic Kaposi’s sarcoma presenting only on the glans penis. Lands, et al. (40)	N/A	Radiation therapy	NO	No recurrences after 1.5 months	N/A
Radiation therapy for classic Kaposi’s sarcoma presenting only on the glans penis. Lands, et al. (40)	N/A	Radiation therapy	NO	N/A	N/A
Kaposi sarcoma limited to the glans penis. Myslovaty, et al. (41)	N/A	Local excision	NO	No recurrences after 6 months	N/A
Primary classic Kaposi’s sarcoma of glans penis - appearance on magnetic resonance imaging. Guy, et al. (42)	N/A	Local excision	YES	Onset of new lesions on the lower extremities after 2 years	N/A
Purplish penile papule as a presenting sign of Kaposi’s sarcoma. Grunwald, et al. (43)	N/A	Local excision	NO	No recurrence after 2 years	N/A
Kaposi’s sarcoma limited to the penis treated with cobalt-60 radiotherapy. Ruszczack, et al. (44)	N/A	Radiation therapy	NO	N/A	N/A
Primary Kaposi’s sarcoma of the glans penis. Koyuncuoglu, et al. (45)	N/A	Local excision	NO	N/A	N/A
Adult genitourinary sarcomas: a report of seventeen cases and review of the literature. Berkmen, et al. (46)	N/A	Local excision	YES	Onset of three new lesions on the shaft after 1 year	N/A
Adult genitourinary sarcomas: a report of seventeen cases and review of the literature. Berkmen, et al. (46)	N/A	Local excision + chemotherapy	YES	Persistence of slight oedema after 1 year	N/A
A case of classical Kaposi’s sarcoma of the penis showing a good response to high energy pulsed carbon dioxide laser therapy. Chun, et al. (47)	N/A	CO2 laser therapy	YES	Onset of a new lesion on the dorsum of the left hand after 5 months	N/A
Penile Kaposi’s sarcoma preceded by chronic penile lymphoedema. Schwartz, et al. (48)	N/A	Local excision + radiation therapy	N/A	N/A	N/A
Penile Kaposi’s sarcoma in a human immunodeficiency virus-seronegative patient. Kavak, et al. (49)	N/A	Local excision	N/A	N/A	N/A

## CONCLUSION

New-onset apparently benign lesions of penis in immunocompetent patients, even in absence of risk factors for sexually transmitted diseases, should be always investigated, because it could represent the first manifestation of primary KS in which penis could be the only isolated clinical presentation. The surgical management could represent a good therapeutic option, leading to disease clinical resolution with no further recurrence, thus providing histological diagnosis.
